# microRNA: Diagnostic Perspective

**DOI:** 10.3389/fmed.2015.00051

**Published:** 2015-08-03

**Authors:** Omar Faruq, Andrea Vecchione

**Affiliations:** ^1^Division of Pathology, Department of Clinical and Molecular Medicine, Faculty of Medicine and Psychology, Ospedale Santo Andrea, Sapienza University of Rome, Rome, Italy

**Keywords:** micro-RNA, biomarkers, diagnosis

## Abstract

Biomarkers are biological measures of a biological state. An ideal marker should be safe and easy to measure, cost efficient, modifiable with treatment, and consistent across gender and ethnic groups. To date, none of the available biomarkers satisfy all of these criteria. In addition, the major limitations of these markers are low specificity, sensitivity, and false positive results. Recently identified, microRNAs (miRNAs) are endogenous, evolutionarily conserved small non-coding RNA (about 22–25 nt long), also known as micro-coordinators of gene expression, which have been shown to be an effective tools to study the biology of diseases and to have great potential as novel diagnostic and prognostic biomarkers with high specificity and sensitivity. In fact, it has been demonstrated that miRNAs play a pivotal role in the regulation of a wide range of developmental and physiological processes and their deficiencies have been related to a number of disease. In addition, miRNAs are stable and can be easily isolated and measured from tissues and body fluids. In this review, we provide a perspective on emerging concepts and potential usefulness of miRNAs as diagnostic markers, emphasizing the involvement of specific miRNAs in particular tumor types, subtypes, cardiovascular diseases, diabetes, infectious diseases, and forensic test.

## Introduction

The ideal biomarker should be non-invasive, specific, cost efficient, quantifiable, robust, translatable, predictive, and sensitive. Physicians, Scientists, and epidemiologists use biomarkers for monitoring, screening, diagnostics, and prognostics of various diseases (1). It is established that disease-related biomarkers including proteins (antigens, enzymes), lipid, and electrolyte components, could be segregated from cells or tissues with current disease and exist in the blood (2). Current available serum/plasma biomarkers are mostly based on proteins. The major limitations of these markers are low specificity, sensitivity, and false positive results. One example among many is given by the serum prostate-specific antigen (PSA), which has been used for screening men with an existing diagnosis of prostate cancer (PCa) and is used as a marker for identifying recurring disease subsequent to treatment. However, PSA-based screening is also associated with over diagnosis and overtreatment. The fact that PSA is synthesized by all prostate epithelial cells, whether normal, hyperplastic, or cancerous, weakens the specificity of PSA as a cancer biomarker. Elevated serum PSA levels may reflect the presence of cancer or may be caused by benign prostatic hyperplasia (BPH), infection, and chronic inflammation (3).

microRNAs (miRNAs) are endogenous, evolutionarily conserved, naturally abundant, relatively stable, small (about 22 nt long) non-coding RNA molecules that function as post-transcriptional gene regulation in most biological and pathological process (4, 5).

It has been estimated that over 30% of protein-coding genes are regulated by miRNAs either by complementary binding of target mRNA or binding to imperfect complementary sites in the 3′ untranslated regions (3′UTR), leading to control of expression of these genes at the level of translation (4, 6, 7). It has been also calculated that above 45,000 miRNAs target sites are present in the human 3′UTR and above 60% of human protein-coding genes are probably regulated by multiple miRNAs (8). miRNAs are transcribed in the nucleus as a long, capped, poly-adenylated precursor called primary miRNA (pri-miRNA) by a RNA polymerase II or III (9, 10). This pri-miRNA is processed by the ribonuclease (RNase) III called Drosha or the double-stranded DNA-binding Protein called DGCR8/Pasha (11) producing a precursor miRNA (pre-miRNA). The nuclear export receptor exportin 5/Ran GTP actively transports pre-miRNAs to the cytoplasm (12, 13). Then, Pre-miRNAs are processed by the RNase III endonuclease Dicer along with the trans-activation-responsive RNA-binding protein (TRBP) and produced a small double-stranded RNA structure (22 nt). This duplex miRNA is unwound into mature single-stranded form and integrate into the RNA-induced silencing complex (RISC), which escorts the complex into the complementary 3′UTR of the target mRNA.

In 2008, circulating miRNAs were identified in blood plasma, platelets, leukocytes, and erythrocytes. Even in the adverse conditions (high or low PH, boiling, freeze–thaw cycle, and long-term storage at room temperature), plasma miRNAs were found to be stable (14, 15). Recently, several studies suggested that plasma miRNAs may be packaged into the exosomes, microvesicles, and apoptotic bodies or associated with the RNA-binding proteins (Arogonaute2) or lipoprotein complex (HDL) to protect their degradation from RNase. Circulating miRNA may mediate distant cell–cell communication, probably to regulate gene expression (16, 17).

Most interestingly, circulating miRNAs have many necessary features of ideal biomarkers. Stable miRNAs have been found in various biological fluids, such as plasma, serum, saliva, milk, cerebrospinal fluids, and markedly expression motifs of serum miRNAs, are firmly linked to various diseases including cancer. The quantity of miRNAs can be easily estimated by various methods, such as Microarray, Hybridization, Deep-sequencing, qRT-PCR, and microbeads analysis.

microRNAs have been demonstrated to play a major role in a wide range of developmental processes including metabolism, apoptosis, cell proliferation, stem cell division, muscle differentiation, and brain morphogenesis (18–20). Since this pivotal role in the regulation of these processes related to development and other physiological functions, miRNAs deficiencies have been linked to a number of diseases ranging from cancer to autoimmune disease.

The aim of this review is to emphasize the great potential of circulating miRNAs as very specific and sensitive biomarkers for diagnosis of tumor and other diseases.

## miRNAs in Diagnosis of Cancer

During the present time, circulating miRNAs are going to the new era of cancer diagnosis and prognosis. Anomalous levels of extracellular miRNAs have been assessed in several cancers, such as hematological and solid neoplasms (summarized in Table 1).

**Table 1:** **Circulating microRNAs a potential biomarkers in human cancers**.

Cancer type	miRNA	Source	Expression	Significance	Reference
Hematological cancer	miR-21	Serum	Up	Diagnosis of DLBCL disease	(21)
	miR-150, miR-342	Plasma	Up	AML vs. healthy controls	(22)
	miR-155	Plasma	Up	CLL vs. healthy controls	(23)
Lung cancer	miR-25, miR-223	Serum	Up	Diagnosis of NSCLC disease	(15)
	miR-1254, miR-574-5p	Serum	Up	Diagnosis of early stage NSCLC patients vs. controls	(24)
	miR-155, miR-197, miR-182	Plasma	Up	Diagnosis of in stage-I NSCLC disease	(25)
Prostate	miR-375, miR-141	Plasma	Down	PCa patient vs. BPH controls	(26)
	MiR-107, miR-574-3P	Urine	Up	PCa vs. healthy controls	(27)
	miR-205, miR-214	Urine	Down	PCa vs. healthy controls	(28)
Pancreatic	Index I (4 miRNAs) and Iindex II (10 miRNAs)	Whole blood	Differential	Early detection of pancreatic cancer vs. healthy controls	(29)
Hepatocellular carcinoma	miR-16, miR-199a	Serum	Down	HCC vs. chronic liver disease. miR-16 with AFP and AFP-L3 tumor protein markers increased sensitivity and specificity of HCC diagnosis	(30)
	MiR-15b, miR-130b, and miR-16	Serum	Up	HCC discriminate HBV hepatitis patients, and also sensitive rather than other tumor biomarkers for diagnosis of HCC	(31, 32)
	miR-625, miR-618, miR-532, miR-650, miR-516-5p	Urine	Differential	HCC (HCV positive) group vs. healthy controls	(33)
Colorectal cancer	miR-29a, miR-92, miR-601, miR-760	Plasma	Differential	Early diagnosis of CRC vs. healthy controls	(34, 35)
	miR-31, miR-181b, miR-92, miR-203 and miR-21, let-7g	Serum	Differential	Good biomarkers for diagnostic of CRC compared with single protein biomarkers	(36)
	miR-200c	Serum	Up	Diagnosis of CRC lymph node metastasis	(37)
Gastric cancer	miR-18a	Plasma	Up	GC vs. healthy controls	(38)
	miR-122, miR-192	Plasma	Differential	Diagnosis of distant metastasis in GC disease	(39)
Ovarian cancer	miR-205, miR-let-7f	Plasma	Differential	Epithelial ovarian cancers (EOC) vs. healthy controls	(40)
Esophageal	miR-21	Serum	Up	Esophageal cancer and BTC vs. controls	(41, 42)
Cervical	miR-218	Serum	Down	Diagnosis of cervical adenocarcinoma and lymphatic metastasis	(43)
Thyroid	let-7f, miR-151-5p, miR-222	Serum	Up	Diagnosis of papillary thyroid cancer (PTC)	(44)
Astrocytomas	miR-15b*, miR-23a, miR-133a, miR-150*, miR-197, miR-497, miR-548b-5p	Serum	Down	Diagnosis of malignant astrocytomas with specificity and sensitivity	(45)
Head and neck	miR-125a, miR-200a	Saliva	Down	Oral squamous cell carcinoma patients vs. control subjects	(46)
Bladder	miR-125b and mir-126	Urine	Differential	Diagnosis of bladder cancer	(46)

*PCa, prostate cancer; BPH, benign prostatic hyperplasia, HCC, hepatocellular carcinoma; CRC, colorectal cancer; GC, gastric cancer; BTC, biliary tract cancer*.

In 2008, Lawrie et al. showed that levels of serum miRNA-21were associated with diagnosis and prognosis in diffuse large B-cell lymphoma (DLBCL) patients (21). More recently, Chen et al. confirmed these results in the sera of Chinese DLBCL patients compared to healthy controls (47). Acute myeloid leukemia (AML) is another type of common hematological cancer in adults. Fayyad-Kazan et al. showed that the expression of two important plasma miRNAs (miR-150 andmiR-342) was up-regulated compared to healthy controls, suggesting that these two miRNAs could be novel, potential, and specific biomarkers for early diagnosis of AML (22). Besides DLBCL and AML, extracellular miRNAs have been also reported in chronic lymphocytic leukemia (CLL). A recent study demonstrated that circulating miR-195, miR-29a, and miR-222 levels are the better indicators to separate B-cell CLL patients from healthy controls (48). In addition, high level of plasma miR-155 seems to be an early detector of the disease (23).

The most common type of lung cancer, non-small cell lung cancer (NSCLC), can be diagnosed in various stages using cell-free non-invasive miRNAs.

Recently, two validated serum miRNAs (miR-25 and miR-223) have been reported to be involved in the tumorigenic process and significantly increased in NSCLC patients, suggesting their potential use in early diagnosis of this lethal neoplasm (15).

Different studies focused on the identification of plasma and serum miRNAs signatures, which could exploit as early stage diagnostic markers for NSCLC. For example, serum-based two miRNAs panel (miR-1254, miR-574-5p) is significantly increased in early stage NSCLC (24), and a plasma based three miRNAs panel (miR-155, miR-197, miR-182) is notably increased in stage-I NSCLC (25). A plasma based four miRNAs panel (miR-21, miR-126, miR-210, miR-182) is differentially expressed in stage-I NSCLC; and furthermore, this panel can be used for differentiate lung adenocarcinoma from squamous cell cancer (49). Lastly, a serum-based 10 miRNAs panel (miR-20a, miR-24, miR-25, miR-145, miR-152, miR-199a-5p, miR-211, miR-222, miR-223, miR-320) is significantly and differentially expressed in early NSCLC patients (50).

Cancer of the PCa is now recognized as one of the most important medical problems facing the male population. Historically, serum PSA has been used for screening men with an existing diagnosis of PCa and has been regarded as an ideal marker for identifying recurring disease subsequent to treatment (51). However, PSA-based screening is associated with over diagnosis and overtreatment (52).

Although Mitchell et al. first reported that serum miR-141 is very sensitive biomarkers to detect PCa, different and contradictory results were obtained by Mahn and co-authors (14, 53). Other studies showed that extracellular miR-375 and miR-141 were most optimistic markers in the diagnosis of this neoplasm (26). Urine, another important source of circulating miRNA, has been widely exploited for the diagnosis of urological cancer. Bryant et al. showed that miRs 107 and 574-3p were significantly higher in the urine of PCa patients compared to healthy groups, and would perform even better than PCA3 normalized to PSA in identifying the presence of PCa (27). Other recent studies reported that urine miR-205 and miR-214 were significantly down-regulated in PCa patients with 80% specificity and 89% sensitivity (28).

Pancreatic cancer is the fourth most common cause of cancer death in the Western world. The prognosis is poor, with 1- and 5-year survival rates of only 20 and 6%. Early diagnosis of pancreatic cancer is difficult, and no biomarkers in blood can identify patients with pancreatic cancer at an early stage. In a recent and promising study, Schultz and colleagues identified two diagnostic panels based on miRNA expression in whole blood with the potential to distinguish patients with pancreatic cancer from healthy controls (29).

Hepatocellular carcinoma (HCC), a form of primary liver cancer, tends to develop in a setting of chronic infection with HBV or HCV viruses, genetic defects, liver injury, or alcohol misuses. Several studies have tried to use extracellular miRNAs for distinguish HCC from other liver disease. Qu et al. reported that serum miR-16 and miR-199a are significant and specifically decreased in HCC compared to chronic liver disease patients. In addition they demonstrated that combining miR-16 with AFP and *Lens culinaris* agglutinin-reactive AFP (AFP-L3) tumor markers could increase sensitivity and specificityin the diagnosisofHCC (30). MiR-15b, miR-130b, and miR-16 are potential, specific diagnostic markers in discriminate between HCC and HBV hepatitis patients, and for early detection of liver cancer as compared to others serum cancer biomarkers such as AFP, des-γ-carboxyprothrombin (DCP), and AFP-L3 (31, 32).

Recently, Abdalla et al. showed that in urine sample, the levels of three miRNAs (miR-625, miR-618, miR-532) are high and levels of two miRNAs (miR-650, miR-516-5p) are very low as compared to the HCC (HCV positive) group and the control groups (33).

Colorectal cancer (CRC) is the third most commonly diagnosed cancer and the third cardinal cause of cancer death in the world. Early diagnosis may risk-free of CRC and can easier target therapy. In several ongoing studies, miRNAs showed to be promising sensitive and specific early detecting molecular biomarkers. Up to date, plasma levels of miR-29a and miR-92 are significantly increased whereas levels of miR-601 and miR-760 are significantly decreased in the early stage of CRC as compared to healthy individuals (34, 35). Recently, Wang et al. reported that inCRC, four cell-free miRNAs (miR-31, miR-181b, miR-92, miR-203) are down-regulated and two miRNAs (miR-21, let-7g) are up-regulated, being more sensitive and specific in the identification of CRC than any single protein biomarker like CEA or CA19-9 (36). Moreover, has been reported that a combination of seven exosomal miRNAs (let-7a, miR-23a, miR-21, miR-150, miR-223, and miR-1229) might be used for early detection of CRC, being significantly increased as compared to healthy groups. In addition, high levels of serum miR-200c in CRC patients have been reported to have a powerful interrelationship with lymph node metastasis (37).

Gastric cancer (GC) is another leading cause of cancer death in the world. For GC diagnosis, fewer numbers of sensitive and specific markers are available. Several studies showed that many extracellular miRNAs are promising and sensitive specific biomarker for early diagnosis of GC (54). More recently, Tsujiura et al. reported that plasma levelsof miR-18a are significantly increased in GC patients (38). In addition, a five miRNAs panel (miR-16, miR-25, miR-92a, miR-451, and miR-486-5p) was identified in plasma as a potential biomarker for early diagnosis of GC (55). Moreover, another study showed that the plasma miR-122 was down-regulated and plasma miR-192 was up-regulated in GC patients and might be used for the early diagnosis of distant metastasis (39).

Briefly, circulating miRNAs have been proven to be useful diagnostic/prognostic markers in other tumors. A two miRNAs signature (high levels of miR-205 and low levels of miR-let-7f) in the plasma of epithelial ovarian cancer (EOC) patients could be able to identify with high specificity and sensitivity EOC patients eventually stage-I disease (40), improving their chances of therapeutic intervention.

Many individual circulating miRNA showed significant association with early cancer diagnosis’s, such as miR-21 levels, that increase in esophageal cancer patients and interestingly together with ROS analysis could be diagnostic for biliary tract cancer (BTC), miR-210 levels are increased in conventional renal cell carcinoma (cRCC) patients, miR-218 levels are significantly reduced in advance stage cervical cancer, cervical adenocarcinoma, and lymphatic metastasis (41–43, 56, 57). In addition, a three miRNAs panel (let-7f, miR-151-5p and miR-222) are significantly up-regulated in sera of papillary thyroid cancer (PTC) patients (44). Moreover, a seven miRNAs panel (miR-15b*, miR-23a, miR-133a, miR-150*, miR-197, miR-497, and miR-548b-5p) is notably reduced in astrocytomas patients, with high sensitivity (88.00%) and specificity (97%) (45).

Furthermore, saliva two miRNAs (miR-125a, miR-200a) were found in reduced in oral squamous cell carcinoma patients. In addition, two validated urine miRNAs (miR-125b and mir-126) are indicated as, highly sensitive (80%), specific (100%), and non-invasive biomarker for bladder cancer diagnosis and prognosis (46).

## miRNA Signatures Can Differentiate between Tumor Subtype

Formalin-fixed paraffin-embedded (FFPE) tissues are a very valuable source for biomarker discovery and validation. Quantity, quality, and the integrity of mRNA into FFPE tissues are not optimal due degradation or cross-linking with proteins and various chemical (58, 59). miRNAs are small size and potentially more stable to degradation than mRNAs during the formalin fixing procedures. Recently, many studies supported and proved that miRNAs are minimally affected by FFPE and, for example, can be simply detected by qRT-PCR in archived liver and CRC specimens up to 30 and 10 years, respectively (60–62).

Identification of tumor subtype is instrumental for patient treatment and therefore survival and the peculiar patterns of miRNAs expression in the individual type of tumor can boost this classification (Table 2).

**Table 2:** **miRNA for tumors sub-typing**.

Cancer	Specific miRNA	Sources	Expression	Significance	Reference
Breast cancer	miR-29a, miR-181a, miR-223, miR-652	Blood	Down		(63)
	miR-29a, miR-181a, miR-652	Blood	Up	
	miR-342	Tissue	Up	Luminal B-like breast tumors vs. healthy controls	(64)
	miR-18a, miR-135b, miR-93, miR-155	Different	Differential	Basal-like vs. healthy controls	(65, 66)
	miR-145, miR-99a, miR-100, miR-130	Different	Differential	Normal-like vs. healthy controls	
Renal cell carcinoma	miR-195, miR-15b, miR-99a, miR-191	Tissue	Up	ccRCC vs. compared with other subtypes	(67)
	miR-200b	Tissue	Up	Chromophobe RCC (chRCC) compared with oncocytoma	(67)
	miR-211	Tissue	Up	chRCC and oncocytoma vs. ccRCC and pRCC	(67)
	miR-210	Tissue	Up	Conventional RCC (cRCC) compared with healthy controls	(56)
Lung Cancer	MiR-21, miR-29b	Tissue	Up	NSCLC vs. SCLC	(68)
	miR-21, miR-126, miR-210, miR-182	Serum	Differential	Diagnosis of stage-I NSCLC and also used in lung adenocarcinoma vs. squamous cell cancer	(49)
	miR-205	Tissue	Up	Squamous vs. non-squamous NSCLC	(69)
Pancreatic Cancer	mR-375	Plasma	Up	Pancreatic ductal adenocarcinoma (PDAC) vs. others pancreatic and gastric carcinoma	(57)
DLBCL	miR-21, miR-155 and miR-221	different	Up	ABC-DLBCL vs. GCB-DLBCL subtypes	(70)
	miR-10393-3p	Tissue	Up	Diagnosis of DLBL patients	(71)
	miR-10397-5p, NOVELMOO288M	Tissue	Up	ABC-DLBCL vs. other DLBCL	(71)
	miR-5586-5p	Tissue	Up	GCB-DLBCL vs. ABC-DLBCL and unclassified DLBCL	(71)
	miR-30b	Tissue	Down	GCB-DLBCL vs. other DLBCL	(71)
	miR-515-3p, miR-155, miR-598, miR-625 miR-199a-5p	Tissue	Differential	PTCL-NOS vs. ALCL/ALK-	(72)
	miR-652, miR-627, miR-519e, miR-487b, miR-324-5p, miR-449a, miR-381, miR-574-3p	Tissue	Differential	PTCL-NOS vs. AITL	(72)
	miR-124, miR-325, miR-181a, miR-618	Tissue	Differential	ALCL/ALK− vs. ALCL/ALK+	(72)
PTCL	miR-512-3p, miR-886-5p, miR-886-3p, miR-708, miR-135b	Tissue	Up	Diagnosis of ALCL/ALK+ disease vs. ALCL/ALK− and other PTCLs	(73)
	miR-146a, miR-155	Tissue	Down	
	miR-210, miR-197, miR-191, miR-512-3p	Tissue	Up	Diagnosis of ALCL/ALK− vs. ALCL/ALK+ and other PTCLs	(73)
	miR-451, miR-146a, miR-22, miR-455-3p, miR-455-5p, miR-143, miR-494	Tissue	Down	

*ccRCC, clear cell renal carcinoma; chRCC, chromophobe renal cell carcinoma; NSCLC, non-small cell lung cancer; SCLC, small cell lung cancer; PDAC, pancreatic ductal adenocarcinoma; DLBCL, diffuse large B-cell lymphoma; ABC, activated B cell-like; GCB, germinal center B cell like; PTCL, peripheral T-cell lymphoma; PTCL-NOS, PTCL-not otherwise specified; AITL, angioimmunoblastic T-cell lymphoma; ALCL, anaplastic large-cell lymphoma; ALCL/ALK+, ALCL− anaplastic lymphoma kinase present; ALCL/ALK−, ALCL− anaplastic lymphoma kinase absent*.

Breast cancer is a highly heterogeneous disease with different molecular alteration, which result in its classification into five intrinsic subtypes (luminal A and B, HER2-enriched, basal-like and normal-like) (75, 76). The original molecular classification is based on investigations on frozen tissue, and it is not applicable to FFPE material, jeopardizing the wide application in the clinical practice. Recently, several studies reported that deregulation of miRNAs expression was strongly associated to molecular breast cancer subtype. In fact, in plasma, the combination of our (miR-29a, miR-181a, miR-223, miR-652) down-regulated and three (miR-29a, miR-181a, miR-652) up-regulated miRNAs was significantly and reliably associated with luminal A tumor (63). Tissue miRNA-342 was highly expressed in luminal B tumor and regarded as a very sensitive and specific biomarker this type of breast cancer (64). Moreover, other miRNAs have been identify to distinguish the remaining subtypes, such as basal-like (miR-18a, miR-135b, miR-93, and miR-155), HER2 type (miR-142-3p, and miR-150), and normal-like (miR-145, miR-99a, miR-100, miR-130) (63–66, 75, 76).

Renal tumors have different genetic backgrounds, prognoses, and responses to surgical and medical treatment, and their differential diagnosis is a frequent challenge for pathologists. Recently, Youssef et al. developed a classification system that can identify the different renal cell carcinoma (RCC) among four subtypes [RCC clear cell RCC (ccRCC), papillary RCC (pRCC), oncocytoma and chromophobe RCC (cRCC)] using a tissue-specific miRNAs signature analysis. This method showed a sensitivity of 97% in discriminating normal from RCC patients, 97% for pRCC from chRCC and oncocytoma, 100% for the ccRCC subtype vs. others subtype and chRCC vs. oncocytoma (67).

The remarkably heterogeneous nature of lung cancer has become more apparent over the last decade. In general, advanced lung cancer is an aggressive malignancy with a poor prognosis. Each subtype is associated with molecular tests that define the subtype and drugs that may potentially treat it.

Gilad et al. proved that eight miRNAs have high accuracy (overall 94%), and high sensitivity (overall 96%) to distinguish among the four subtypes of lung cancer [small cell lung cancer (SCLC), NSCLC, squamous cell carcinoma and carcinoid] (77). Hsa-miR-21 and miR-29b are expressed at higher levels in NSCLC versus SCLC and miR-129-3p, miR-205 can be utilized to differentiate squamous from non-squamous NSCLC (68, 69).

Diffuse large B-cell lymphoma is a common form of high-grade non-Hodgkin lymphoma (NHL) that account for 30–40% of all lymphoid malignancies. DLBCL includes a biologically and clinically diverse set of diseases, many of which cannot be separated from one another by well-defined criteria.

Many studies now suggest that DLBCL can be separated into two groups on the basis of their gene expression profiles: activated B cell-like (ABC) and germinal center B cell like (GCB). Recently, miRNAs have been used for characterization of DLBCL subtypes. Lawrie et al. reported that three miRNAs (miR-21, miR-155, and miR-221) are significantly expressed in DLBCL cases of ABC subtype as compared with GCB (70). Lim et al. demonstrated that miR-10393-3p is significantly over-expressed in DLBCL patients (71). Moreover, two miRNAs (miR-10397-5p, NOVELMOO288M) can distinguish ABC-DLBCL patients from other DLBCL patients (71). Furthermore, miR-5586-5p can be used for distinguish GCB-DLBCL from ABC-DLBCL, and miR-30b can also be used to discriminate GCB-DLBCL from other DLBCL disease group (71).

Peripheral T-cell lymphoma (PTCL) is a NHL, which refers to a diverse group of uncommon and aggressive nodal, extra nodal, and leukemic tumors that represent about 10–15% of all lymphoid neoplasm. The most common subtypes of PTCL are PTCL not otherwise specified (PTCL-NOS), angioimmunoblastic T-cell lymphoma (AITL), anaplastic large-cell lymphoma (ALCL) anaplastic lymphoma kinase present (ALK+), and ALCL/ALK− (72, 78). Specifically, PTCL-NOS is a rare and life-threatening disease whose differential diagnosis from AITL and ALCLs are still challenging. Most recently, Laginestra et al. validated a five miRNAs set (miR-515-3p, miR-155, miR-598, miR-625, miR-199a-5p) that can differentiate PTCL-NOS from ALCL/ALK−, a eight miRNAs panel (miR-652, miR-627, miR-519e, miR-487b, miR-324-5p, miR-449a, miR-381, miR-574-3p) that can be used to distinguish PTCL-NOS from AITL, and also a four miRNAs signature (miR-124, miR-325, miR-181a, miR-618) that can be used to identify ALCL/ALK− from ALCL/ALK+ disease group (72). Liu et al. confirmed two miRNA signatures that can be used as a diagnostic tool for biological difference of PTCLs sub-typing, and can play a crucial role in pathogenesis. In the first miRNA signature, the levels of five miRNAs (miR-512-3p, miR-886-5p, miR-886-3p, miR-708, miR-135b) are up-regulated, and levels of two miRNAs (miR-146a, miR-155) are down-regulated as compared to ALCL/ALK+ disease group from ALCL/ALK−, and other PTCLs disease group (73). Moreover, the second miRNA signature is used to differentiate ALCL/ALK− from ALCL/ALK+, and other PTCLs disease group (Table 2) (73).

## miRNAs in Diagnosis in Other Diseases

Cardiovascular diseases (CVD) still represent the primary cause of morbidity and mortality worldwide. The understanding and the identification of CVD molecular factors that may significantly reduce the patient death rates still remain a challenge (74). Protein biomarkers, such as cardiac troponin (cTn), CK-MB, brain natriuretic peptide (BNP), and N-terminal prohormone (NT-proBNP), have been used to identify and monitor myocardial damage. Unfortunately, these biomarkers provided false positive results and are also time-dependent (79, 80). Therefore, for early diagnosis of cardiac damage other types of biomarker are the need of the hour. miRNAs have been proven to have an important role in CVD, including acute myocardial infarction (AMI), hypertrophy, heart failure, arrhythmias, and atherosclerosis (81). Tijsen et al. demonstrated that blood miR-423-5p was highly expressed in heart failure patients (independent of age and gender) and might represent a significant and sensitive biomarker for heart disease (82). Recently, it has been reported that elevated plasma levels of this miR are positively linked with NT-proBNP levels in dilated cardiomyopathy heart failure patients (83).

D’Alessandra et al. reported that plasma levels of miR-1, miR-133a/b were elevated 156 ± 72 min after the onset AMI symptoms; whereas plasma levels of miR-122 and miR-375 were lower only ST-segment elevation myocardial infarction (STEMI) patients than in healthy controls (84). Other studies showed that plasma levels of miR-21, miR-1, miR-26, miR-133, miR-30, miR-328, and miR-499 were significantly expressed in atrial fibrillation (85).

Takotsubo cardiomyopathy (TTC), a type of non-ischemiccardio myopathy unrelated to AMI, is a major life-threatening condition. To date, there are not early biomarker for its diagnosis and its distinction from AMI. Jaguszewski et al. reported that a unique signature comprising miR-1, miR-16, miR-26a, and miR-133a is capable of distinguish TTC from healthy individuals (sensitivity 74.19%, specificity78.57%) and from AMI patients (sensitivity 96%, specificity70.37%) (86).

Diabetes mellitus (DM) is a metabolic shuffle identified by hyperglycemia-mediated life-threatening complications. Many studies showed that circulating miRNAs are linked with type 1, 2, and gestational DM and its complications. Levels of circulating miR-21 and miR-210 were significantly high in the plasma and urine of the type 1 diabetic patients, while in urine miR-126 was notably lower in type-1 diabetic patients compare to controls (87). Peng et al. showed that the levels of urinary miR-29a were significantly high in albuminuria with type 2 DM than in healthy controls and urinary miR-29b was correlated with carotid intima-media thickness (cIMT) in patients with type 2 diabetes (88). In addition, the levels of platelet and plasma miR-223 and miR-146a were significantly reduced in patients with ischemic stroke and DM or only DM (89). Recently, Luo et al. demonstrated that platelet-derived miR-103b was specifically, down-regulated in early type 2 DM (90).

HIV/AIDS is a spectrum of conditions caused by infection with the human immunodeficiency virus type 1 and 2. For diagnosis of HIV/AIDS, several types of tests can help the physician to determine the patients HIV infection status, to calculate the evolution of the disease, and to monitor the efficacy of anti-retroviral therapy. Several studies demonstrated that host–virus interactions (including HIV) are controlled, at least in part, by miRNAs and these might lead to new diagnostic and attractive therapeutic strategies. Recently, Monteleone et al. proved that plasma miR-29b was highly expressed in HIV-1 patients, while lower levels of plasma miR-29cindicated high viremia, low CD4+ T cell count and high levels of integrated HIV-1 DNA, suggesting that the miR-29 family could be implicated in the natural history of HIV-1 infection (91). Another group reported that plasma levels of two miRNAs (miR-150 and miR-146b-5p) also varied between different groups ofHIV patients and between patients and healthy individuals (92). Moreover, expression levels of miR-150 in peripheral blood mononuclear cells (PBMCs) and plasma might be a better and specific biomarker for diagnosis of the different status of HIV/AIDS disease. MiR-150 levels were decreased in PBMCs but increased in plasma of HIV patients and drug-resistant HIV patients. On the other hand, miR-150 levels were increased in PBMCs and decreased in plasma of anti-retroviral therapy patients (92). In addition, Sekar et al. reported that miR-21 over-expression is considerably associated with the onset of HIV-related lymphomas (93). Furthermore, the levels of extracellular miR-122 were highly expressed in HIV/HCV co-infected patients (94).

## Use of miRNAsin Forensic Diagnostic

Isolating the origin of body fluids in a crime scene would be instrumental for its reconstruction; however, conventional protein based methods for body fluid identification are prone to various limitations, such as sample consumption, varying degrees of sensitivity and specificity, and for mRNA based markers, stability.

The shorter lengths of miRNAs make them less susceptible to degradation caused by chemical and/or physical environmental factors, thus rendering them useful biomarkers for body fluid identification.

Hanson et al. showed the expression of miRNAs in dried forensically relevant biological fluids: blood, saliva, menstrual blood, and vaginal secretion. They distinguished nine panels of miRNAs that are differentially expressed and can be used as sensitive biomarkers to identify the origin of body fluid of forensic biological stains (95) (Table 3).

**Table 3:** **Circulating miRNAs a potential diagnostic biomarkers in other disease**.

Disease	miRNA	Source	Expression	Significance	Reference
Cardiovascular disease	miR-423-5P	Blood	Up	Diagnosis of heart failure patients compared to healthy controls	(74, 73)
	miR-1, miR-133a/b	Exosomes	Up	Diagnosis of AMI, and biomarker for cardiomyocyte death disease	(79)
	miR-122 and miR-375	Plasma	Up	STEMI patients vs. healthy controls	
	miR-21, miR-1, miR-26, miR-133, miR-30, miR-328, and miR-499	plasma	Up	Diagnosis of arterial fibrillation specific heart disease vs. healthy controls	
	miR-1, miR-16, miR-26a, and miR-133a	Sera	Up	TTC vs. healthy controls	(81)
Diabetic mellitus	miR-21 and miR-210	Urine and plasma	Up	Type-1 diabetic patients vs. healthy controls	(82)
	miR-29a	Urine	Up	Albuminuria with type2 diabetes mellitus vs. healthy controls	(83)
	miR-223 and miR-146a	Plasma, platelet	Down	Diagnosis of ischemic stroke and diabetes mellitus or only diabetes mellitus compared to controls	(84)
	miR-130b	Blood	Down	Diagnosis early type 2 diabetes mellitus	(85)
HIV/AIDS	miR-29b	Plasma	Up	HIV1 patients vs. controls	(86)
	miR-29c	Plasma	Down	High viremia, low cd4+ T cells	
	miR-150 and miR-146b-5p	Plasma	Differential	HIV patients vs. controls	(87)
	miR-21	Plasma	Up	Diagnosis of HIV-related lymphomas	(88)
	miR-122	Plasma	Up	Diagnosis HIV/HCV co-infection	(89)
Forensic	Nine miRNA panel (miR-451, miR-16, miR-135b, miR-10b, miR-658, miR-205, miR-124a, miR-372, miR-412)	Blood, semen, saliva, vaginal secretions menstrual blood	Differential	Sensitive biomarkers identification of forensic biological stains	(90)

*AMI, acute myocardial infarction; STEMI, ST-segment elevation myocardial infarction; TTC, Takotsubo cardiomyopathy*.

## Experimental Techniques and Normalization of Circulating microRNAs

Accurate measurement of tissue and body fluid miRNAs has clinical importance for diagnosis and prognosis of several types of disease. miRNAs detection is a very challenging task for their native properties, e.g., small size, scarce concentration, high risk of cross-hybridization, and tissue/stage-specific expression. Therefore, quantification of cell-free miRNAs needs powerful tools. The rapid improvement of diagnostic device platforms is one of the leading experimental goals in the world of life science research and medical diagnostics. The explosion of high interest in miRNAs, over the recent years, brought to the development of many platforms. High-throughput miRNA profiling techniques including qRT-PCR, microarray, deep sequencing or next generation sequencing (NGS), and nanopores are effective tools for obtaining expression profiles of extracellular miRNAs. These techniques highly smoothen the process of free cell miRNA expression profiling. In this view, high-throughput techniques are better to the existing low-throughput techniques such as northern bolting and *in situ* hybridization (ISH) (96). Currently, qRT-PCR is the most common, reliable and available, inexpensive method used for quantifying the small amount of miRNAs with the high sensitivity and specificity and the subsequent validation of clinical samples (96, 97). However, some limitations of qRT-PCR, such as normalization of data and primer design, can highly influence the results. The lack of standardized housekeeping RNA for normalization, especially in body fluid, is a critical issue (97). Many miRNAs can be used as control reference such as miR-16, miR-142-3p, miR-30b, miR-145, miR-93, U6, U6B, SNORD68, RNU48, RNU43, RNU62, and 5sRNA. Kirschner et al. reported that miR-16 is highly expressed in red blood cells and that its levels in the extracellular content of blood fluids can differ significantly due to hemolysis (98). Moreover, many studies suggested that several endogenous genes might be diseases specific. Therefore, some miRNAs, such as miR-16 and miR-93 and SNORD68, might be considered satisfactory reference gene for miRNAs serum analysis of gastric and urologic cancers, respectively (99, 100). At present, there is no unique housekeeping RNA that can be used as the standard endogenous reference gene in normalizing circulating miRNAs. Currently, many studies suggest the use of more than one reference genes or better a combination for normalizing circulating miRNA concentrations.

## Perspective

An optimal biomarker should be sensitive, specific, non-invasive, and comparable to the disease conditions. In the new decade, many studies clearly proved that cell-free, miRNAs, and tissue miRNAs fulfill these expectations.

Despite the encouraging progress in the practical areas, several major theoretical and technical difficulties remain to be addressed. First, more basic investigations will be required to further examine the functional role of miRNAs in cancer, cardiovascular, and other disease. Although, miRNAs can inhibit mRNAs by translational repression or cleavage, others possible unknown mechanism could lead to translational or post-translation repression. Second, the biological functions and packaging of extracellular miRNAs needs to be better elucidated. A limited number of studies proposed that microvesicles containing miRNAs might be involved in the tumor microenvironment. Finally before translation into clinical practice, all circulating miRNA findings require further steps of validation and an accurate standardization of all preanalytical and analytical procedures, in order to control for all potential technical biases.

## Conflict of Interest Statement

The authors declare that the research was conducted in the absence of any commercial or financial relationships that could be construed as a potential conflict of interest.
